# Regional Variation in Use of End-of-Life Care at Hospitals, Intensive Care Units, and Hospices Among Older Adults With Chronic Illness in the US, 2010 to 2016

**DOI:** 10.1001/jamanetworkopen.2020.10810

**Published:** 2020-07-21

**Authors:** Jason H. Maley, Bruce E. Landon, Jennifer P. Stevens

**Affiliations:** 1 Division of Pulmonary, Critical Care, and Sleep Medicine, Beth Israel Deaconess Medical Center, Boston, Massachusetts; 2 Beth Israel Deaconess Center for Healthcare Delivery Science, Boston, Massachusetts; 3 Division of General Medicine, Beth Israel Deaconess Medical Center, Boston, Massachusetts; 4 Department of Health Care Policy, Harvard Medical School, Boston, Massachusetts

## Abstract

This cohort study examines regional patterns in end-of-life health care use among Medicare beneficiaries with chronic diseases treated at US hospitals, intensive care units, and hospice facilities from 2010 to 2016.

## Introduction

Most older adults living with chronic illness prefer palliation of symptoms at home rather than invasive therapies or hospitalization at the end of life.^1^ In the United States, death occurring at home has become more common than death occurring in the hospital.^2,3^ However, national patterns may overlook important regional variation in end-of-life (EOL) care. Additionally, research is lacking on these patterns among the increasing population of older adults with chronic illness.^1,3,4,5^ Therefore, we sought to assess recent temporal patterns and regional variation in end-of-life health care use by Medicare beneficiaries with chronic illness.

## Methods

This retrospective cohort study was deemed exempt by the Beth Israel Deaconess Medical Center Institutional Review Board, with a waiver of informed consent granted because the data were deidentified and publicly available. The cohort included decedents from 2010 through 2016 with a diagnosis of at least 1 of 9 chronic illnesses most commonly associated with death in the Medicare-eligible population: malignant cancer or leukemia, congestive heart failure, chronic pulmonary disease, dementia, diabetes with end-organ damage, peripheral vascular disease, chronic renal failure, severe chronic liver disease, and coronary artery disease.^6^ This study is reported in accordance with the Strengthening the Reporting of Observational Studies in Epidemiology (STROBE) reporting guideline.

We used data derived from Medicare fee-for-service claims^6^ in the 2 years prior to death to examine hospital referral region (HRR)–level variation and temporal patterns in the percentage of Medicare beneficiaries with chronic illnesses who died in 3 settings: hospital, hospital with intensive care unit (ICU) admission, and hospice. We examined all of the HRRs in the US. HRRs represent health care markets defined by sites of tertiary medical care.

Data were adjusted for age, sex, race, primary chronic condition, and the presence of multiple chronic conditions. A detailed description of the methods is available from the Dartmouth Atlas.^6^ For continuous variables, we computed means with standard deviations or medians with interquartile ranges. Categorical variables are described as numbers and percentages. Analyses were conducted from December 10, 2019, to January 20, 2020, using R, version 3.6.1 (R Project for Statistical Computing).

## Results

We identified 7 425 913 Medicare beneficiaries diagnosed with at least 1 of 9 chronic illnesses most commonly associated with death in the Medicare-eligible population who died from 2010 through 2016. These illnesses included malignant cancer or leukemia, congestive heart failure, chronic pulmonary disease, dementia, diabetes with end-organ damage, peripheral vascular disease, chronic renal failure, severe chronic liver disease, and coronary artery disease. The mean (SD) patient age was 81 (8.4) years, and 4 119 749 (55%) Medicare beneficiaries were women. In 2010, Manhattan, New York, had the highest rate of EOL hospitalization (43.7%), followed by 3 other HRRs in New York state (Bronx, 37.7%; East Long Island, 37.4%; White Plains, 36.0%) (Table). These HRRs also had among the lowest rates of hospice enrollment among a total of 306 HRRs. In 2010, the rate of EOL hospitalization was lowest in Amarillo, Texas (13.3%), Greeley, Colorado (13.5%), and Ogden, Utah (13.9%); these HRRs had among the highest rates of hospice enrollment. In 2016, Manhattan, New York, again had the highest rate of hospitalization at the EOL (34.5%), whereas Cedar Rapids, Iowa, had the lowest rate (11.2%). Therefore, rates of EOL hospitalization and hospice use varied greater than 3-fold across all 306 HRRs. Even among the 20 largest HRRs, rates of hospitalization and hospice at the EOL varied greater than 2-fold between high and low-use regions (Figure). In 2016, among the 20 largest HRRs, rates of EOL hospitalization ranged from 14.7% to 34.5%. Rates of EOL hospice ranged from 33.6% to 68.2%.

**Table.** Health Care Resource Use From 2010 to 2016 Among HRRs With the Highest and Lowest 2010 End-of-Life Hospitalization Rates

HRR	No. of decedents		Hospitalization, % ^a^		Hospitalization with ICU admission, % ^b^		Hospice enrollment, %	
	2010	2016	2010	2016	2010	2016	2010	2016
All HRRs, median (IQR)	1 107 702	1 058 187	23.9 (20.8-27.5)	20.1 (17.5-23.0)	15.8 (13.5-18.2)	14.2 (12.2-16.1)	47.8 (41.0-54.3)	55.5 (49.3-60.6)
HRRs with highest end-of-life hospitalization rate								
Manhattan, New York	12 823	10 125	43.7	34.5	19.4	20.2	23.0	33.6
Bronx, New York	2392	1885	37.7	28.4	19.5	16.6	20.8	28.8
East Long Island, New York	16 466	15 097	37.4	31.1	20.5	18.3	31.4	38.5
White Plains, New York	4502	4197	36.0	31.9	21.9	21.4	28.1	32.5
McAllen, Texas	1669	1060	35.2	25.1	31.0	22.0	27.1	48.5
Elmira, New York	1868	1438	34.2	27.9	17.3	18.8	19.3	26.7
HRRs with lowest end-of-life hospitalization rate								
Amarillo, Texas	2022	1956	13.3	12.0	8.2	8.5	65.0	65.5
Greeley, Colorado	1252	1343	13.5	16.7	9.0	10.4	44.8	47.9
Ogden, Utah	965	1147	13.9	16.1	11.3	12.3	66.5	67.6
Dubuque, Iowa	529	390	15.2	13.7	7.1	8.7	58.4	62.7
Sun City, Arizona	1591	1640	15.5	11.9	13.6	11.2	70.3	71.6
Mason City, Iowa	973	1004	15.5	13.9	7.2	7.6	59.2	62.4

Abbreviations: HRR, hospital referral regions; ICU, intensive care unit; IQR, interquartile range.

^a^ Hospitalization was calculated as the number of patients hospitalized at the end of life (including all hospitalizations regardless of whether there was an ICU admission) divided by all decedents.

^b^ Hospitalization with ICU admission was calculated as the number of patients hospitalized with an ICU admission at the end of life divided by all decedents.

**Figure.**
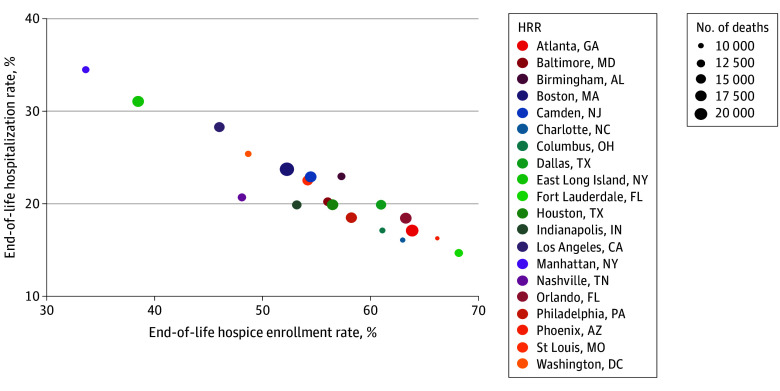
Hospital Referral Region (HRR)–Level Rates of Hospitalization and Hospice Use at the End of Life in the US, 2016 Rates are shown for the 20 hospital referral regions with the largest populations of decedents. Point size is scaled by the total number of decedents in the hospital referral region in 2016.

From 2010 to 2016, the rate of EOL hospitalization decreased in 283 of 306 HRRs (92.5%) (mean change, −4.2% per HRR; range −13.9% to 5.8%), and hospice enrollment increased in 92.9% of HRRs (mean change, 6.9% per HRR; range, −6.7% to 21.4%). Most EOL hospitalizations included an ICU admission. For example, in 2016, 73.2% of EOL hospitalizations in Cedar Rapids, Iowa, and 58.5% in Manhattan, New York, included an ICU admission.

## Discussion

From 2010 through 2016, the rates of hospitalization at the EOL decreased and hospice enrollment increased in nearly all regions in the US. Nonetheless, Medicare beneficiaries with chronic illnesses continue to experience marked regional variation in EOL hospitalization, ICU use, and hospice use—these rates varied more than 3-fold across all US regions. Despite a common preference to avoid EOL hospitalization and intensive therapies, the region of residence may remain an important determinant of the site of EOL care.^1^

This study has important limitations. Many factors may play a role in the observed temporal patterns and HRR-level variation, including changes to health policy and practice, the supply of hospital and hospice services, and health care spending. We did not assess these factors in the current study. We included fee-for-service claims only. Hospice rates include inpatient and home hospice. In addition, unmeasured patient-level covariates may contribute to hospitalization and hospice rates and confound observed HRR-level associations between these rates. Although overall patterns in EOL care are encouraging, future research should focus on identifying modifiable factors that contribute to persistent regional disparities in EOL care such that patients’ values, rather than region of residence, guide the dying experience.
